# Effects of psychedelic drug use on neurocognitive function and the psychological and social domains of quality of life: an international online study

**DOI:** 10.3389/fpsyt.2026.1843186

**Published:** 2026-09-24

**Authors:** Franziska Stadler, Johan Saelens, Ioline D. Henter, Nathalie M. Rieser, Maximillian S. Greenwald, Elizabeth D. Ballard, Katrin H. Preller, Carlos A. Zarate, Christoph Kraus

**Affiliations:** 1 Department of Psychiatry and Psychotherapy, Medical University of Vienna, Vienna, Austria; 2 Comprehensive Center for Clinical Neurosciences and Mental Health, Medical University of Vienna, Vienna, Austria; 3 Experimental Therapeutics and Pathophysiology Branch, National Institute of Mental Health, National Institutes of Health, Bethesda, MD, United States; 4 Department of Adult Psychiatry and Psychotherapy, Psychiatric University Clinic Zurich and University of Zurich, Zurich, Switzerland; 5 Yale University School of Medicine, The Connecticut Mental Health Center, New Haven, CT, United States

**Keywords:** cognition, ketamine, online study, psilocybin, psychedelics

## Abstract

**Background:**

The past few decades have seen a revived interest in psychedelic-assisted and ketamine-assisted therapy for mental health disorders, but data concerning the acute, subacute, and long-term effects of serotonergic psychedelics on cognitive functioning are scarce. This international, online study (N = 759) sought to investigate the potential acute, subacute, and long-term effects of psychedelic drug use on cognition through cognitive task assessment. Questionnaires assessing mental health and quality of life were also administered.

**Methods:**

Participants completed cognitive tasks assessing working memory, selective attention, and visual/spatial perception, as well as questionnaires assessing mental health outcomes and quality of life. Based on self-reported substance use, participants were classified as non-users, lifetime users, and recent users.

**Results:**

Recent users had significantly lower accuracy across all cognitive tasks, and lifetime users had the highest task accuracy without corresponding reaction time deficits, with the exception of the 2D mental rotation task. Lifetime use was not associated with long-term cognitive decline. Recent users reported more depressive and dissociative symptoms, whereas lifetime users reported lower scores. Lifetime users scored lower on psychological and social quality of life domains, indicating possible long-term psychosocial effects.

**Conclusions:**

These findings highlight the need to differentiate between the acute and long-term effects of psychedelics; lab-controlled, longitudinal studies are needed to enable safe clinical application.

## 1 Introduction

The term “psychedelic”, which is not well classified and lacks definition, is often applied specifically to serotonergic 5-HT_2A_ receptor (5-HT2AR) agonists. Some investigators have argued that the term “psychedelic” should be used in a broader sense to include substances with overlapping therapeutic potential, such as ketamine or 3,4-methylenedioxymethamphetamine (MDMA) (1). Psychedelic substances such as psilocybin, lysergic acid diethylamide (LSD), mescaline, N,N-dimethyltryptamine (DMT), 2,5-Dimethoxy-4-iodoamphetamine (DOI), and 5-methoxy-DMT (5-MeO-DMT) act primarily by activating the serotonergic 5-HT2ARs and are referred to as “classic psychedelics” (2–4). More dissociative agents such as ketamine and phencyclidine (PCP) primarily act via glutamatergic pathways (5). The past few decades have seen a revived interest in psychedelic-assisted and ketamine-assisted therapy for mental health disorders (6, 7), and this renewed focus has led these promising treatments to the verge of regulatory approval (8, 9). For instance, esketamine—the *S*-stereoisomer of ketamine—received approval from the United States Food and Drug Administration (FDA) and the European Medicines Agency (EMA) for adults with treatment-resistant depression (TRD) or major depressive disorder (MDD) with suicidal ideation. In addition, the Australian Therapeutic Goods Administration (TGA) introduced a controlled prescribing framework allowing registered psychiatrists to prescribe MDMA for post-traumatic stress disorder under specific regulatory conditions, including mandatory six-month reports on adverse events and patient numbers (10).

These clinical developments are supported by population-level findings on psychedelic use, but questions regarding the safety of these psychoactive substances remain. One population study found no significant association between lifetime use of psychedelics and the prevalence of mental health symptoms but did identify a weak positive correlation with better mental health outcomes (11). Other studies found no impairment in mental health outcomes following psychedelic drug use (12–14). However, to enable more widespread and safe clinical use, potential adverse events need to be better understood, particularly with regard to cognitive functioning (15, 16), which plays a crucial role in psychedelic research due to its overlapping neuronal pathways and its bidirectional relationship with mental health processes. Given that psychedelic substances interact with neural systems implicated in higher-order cognitive processes, there is increasing interest in understanding their potential effects on cognitive domains such as attention, memory, executive functioning, and visual perception (17–25).

Nevertheless, data concerning the acute, subacute, and long-term effects of serotonergic psychedelics on cognitive functioning are scarce. A double-blind, placebo-controlled study that explored the acute effects of a macrodose (100mcg) of LSD on working memory, executive functioning, and cognitive flexibility found acute impairment in all three domains; furthermore, these were not observed when LSD was taken with adjunctive ketanserine, a 5-HT_2A_ receptor antagonist (26). Although two recently published reviews (15, 27) explored the acute effects of psychedelics, few data exist on their long-term effects. Likewise, emerging research regarding the cognitive effects of microdosing (broadly defined as ingesting one-tenth of a full dose) has been inconclusive (28). One double-blind, placebo-controlled study exploring the effects of microdosing LSD found that it improved sustained attention and did not impair cognitive functioning, such as working memory, visual processing, and cognitive control (29, 30). Likewise, other studies investigating the acute effects of microdosing psilocybin or LSD identified no significant impairment or enhancement of cognitive functioning (31–33).

Ketamine, which has hallucinogenic and dissociative properties, and esketamine (ketamine’s *S*-stereoisomer) are glutamatergic N-methyl-D-aspartate receptor (NMDAR) antagonists and have also been associated with changes in cognitive functioning (34–38). In contrast to the dearth of research regarding the acute effects of serotonergic psychedelics on cognition, several studies have examined the acute effects of ketamine. Short-term ketamine use appears to impair both episodic and working memory (39, 40), and chronic ketamine users also appear to have impaired cognitive functioning (working memory, visual memory, and executive functioning) (41). One longitudinal study that assessed memory impairment over the course of one year in recreational frequent users of ketamine alone (~20 out of 30 days/month) found significantly impaired memory processing (36). The study also found that ketamine’s effects were dose-dependent, with higher doses of ketamine leading to greater impairments in cognitive functioning (42). In contrast, an exploratory, double-blind, placebo-controlled study of 21 individuals with TRD found no impairments in working memory, attention, or concentration post-ketamine (43). Another randomized, double-blind, placebo-controlled study of 22 participants with TRD and 19 healthy participants found that a single dose of ketamine had no effect on working memory (44).

In this context, the impact of classic psychedelics and dissociative agents on cognition and mental health calls for further exploration. This international, online study sought to address existing gaps in the data by investigating the potential effects of psychedelics on cognition through cognitive task assessment. Questionnaires assessing mental health and quality of life were also administered. The hypothesis was that lifetime or subacute psychedelic drug use would significantly impact working memory, selective attention, and visual perception.

## 2 Materials and methods

### 2.1 Study design

This was a naturalistic, international online study evaluating the effects of psychedelic drug use on cognitive functioning. It was conducted on the platform Gorilla (www.gorilla.sc), an online experiment builder that allows reliable online measurement of neurocognitive tasks (45, 46). For feasibility reasons, a set of questionnaires and tasks that could be filled out during an approximately 30-minute period was used. Ethical approval was obtained by the Ethical Commission of the Medical University of Vienna, Austria (reference number: 1618/2021), and the study was registered on https://www.osf.io (https://doi.org/10.17605/OSF.IO/N7UHX). Participants gave consent for study design and data usage by agreeing to a consent form at the beginning of the study after the landing page.

### 2.2 Participants and recruitment

The study recruited participants worldwide who were over the age of 18 and who accessed the study via a laptop or PC; the study specifically targeted people interested in the realm of psychedelics. All questionnaires that were complete and carefully filled out in the allotted amount of time were included. There were no specific exclusion criteria apart from minimum age of participation and outcomes with implausible answers or reaction times.

Participants were recruited between February 2022 and December 2024. A landing page was generated where participants could access the study and, if interested, were redirected to gorilla.sc. Recruitment strategies included advertisement on social media platforms (Instagram, Facebook, and Twitter) as well as promotion on the Reddit platform, where participants interested in psychedelics were actively sought in subreddits dedicated to specific topics, such as “psychonaut”, “mushrooms”, “HPPD”, “RationalPsychonaut”, “microdosing”, “psychedelics”, “LSD”, “depressionregimens”, and “PsychedelicTherapy”. The study was also promoted via the third party provider Amazon Mechanical Turk, a platform that links researchers and participants; participants who took part via Amazon Mechanical Turk were financially reimbursed with $5 USD.

### 2.3 Procedure

Participants were first asked for their demographic data as well as mental health conditions, including psychiatric diagnoses, history of inpatient psychiatric care, history of drug or alcohol use, and hospitalization for psychoactive drug-related incidents.

Participants were then questioned regarding their history of psychedelic drug use. Options included serotonergic psychedelics (psilocybin, LSD, DMT, tryptamine (5-MeO-DMT), mescaline, DOI), dissociative agents with psychedelic properties (e.g, the glutamatergic modulators ketamine and PCP), and substances that exert psychedelic effects via different pharmacological mechanisms, such as ibogaine, scopolamine, salvia, and MDMA (47). Data on further drug use, such as alcohol, cocaine, cannabinoids, and opiates were also collected. Participants reported the frequency of use within the past month, the frequency of use during their period of highest use, the number of classic psychedelics and substances with hallucinogenic properties used both in their lifetime and in the past week, and the median dose of each used substance. Age of first use of a psychedelic was also assessed.

### 2.4 Cognitive tasks

Five cognitive tasks were used to assess the three cognitive domains: working memory, selective attention, and spatial/visual perception. The n-back task (in this study a 2-back task), and the digit span text entry task were used to assess working memory. Selective attention was measured using the dot-probe task, which captures attentional shifts in response to visual stimuli (48). For visual and spatial perception, the 2D mental rotation task (49) and the visual search task were used. A more detailed description of the task procedures is available in the Supplement. All tasks were provided by the Gorilla platform (www.gorilla.sc), although trial answers were partly designed by the research team (see Supplement). Task reaction times and number of correct answers were recorded using Gorilla.sc, ensuring timing accuracy comparable to lab-based setups through high-resolution timers and frame counting (45). Responses were given manually on the keyboards of the participants’ devices. Before each task, participants completed a test trial.

Participant data with reaction time less than 200ms or above 10000ms of reaction time were removed only for the task in question (the dot-probe task and the 2-back task had implemented maximum time limits). Only the trial in question was excluded, while the rest of the participant’s data were included in analysis.

### 2.5 Questionnaires

Participants completed four questionnaires concerning mental health and quality of life. Symptoms of depression were assessed using the Patient Health Questionnaire (PHQ-9) (50), overall quality of life and health was evaluated with the World Health Organization Quality of Life questionnaire (WHOQOL-BREF) (51), and dissociative symptoms were measured using the Brief Dissociative Experiences Scale (DES-B) (52). To assess clinical symptoms of Hallucinogen Persisting Perception Disorder (HPPD), a nonvalidated symptom checklist (53) designed by Steven Locke, MD measuring symptoms experienced within the month prior to participation was used. The checklist, which sums 12 items worth 0–3 points each, focuses on distortions of visual perception such as colors, geometric patterns, shapes, and light. Participants responded to each question using a scale with options ranging from “not at all”, “several days”, and “more than half the days” to “nearly every day”.

### 2.6 Statistical analysis

Data were extracted from Gorilla (https://www.gorilla.sc) via spreadsheets and analyzed in Rstudio (version 4.4.1) (54). One participant who did not meet inclusion criteria because they were underage was removed from the dataset. Extreme outliers in the cognitive tasks—those with a reaction time above 10s or lower than 200ms—were also removed for each trial in question (see Supplement).

Demographic data and mental health diagnoses were analyzed using descriptive statistics including mean, median, standard deviation, minimum, and maximum of the metric variables. The participants were divided into three groups: non-users (defined as participants who stated that they had never previously used a psychedelic drug); lifetime users (defined as those who had used at least one psychedelic drug at least once in their lives); and recent users (defined as participants who had taken at least one psychedelic drug in the week prior to taking part in the study). Furthermore, some participants across both groups of users stated that they were under the influence of a psychoactive drug while completing the study tasks (hereafter referred to as “currently-using” users); to test for acute effects associated with psychoactive drug use, this group of “currently-using” users was compared to all users not under the influence of a psychoactive drug regardless of their previous psychedelic drug use history. An exploratory re-analysis was conducted excluding 23 participants in the lifetime group who reported lifetime use and acute psychoactive intoxication during study participation but who also indicated no psychedelic substance use within the week preceding participation (**Supplementary Tables S7**, **S8**). To further address this bias, an additional exploratory analysis was conducted excluding all “currently-using” participants in order to allow a clearer comparison between long-term and subacute effects (**Supplementary Table S9**). It should be noted that while the questionnaire asked: “Are you currently under the influence of a psychoactive drug while completing the questionnaire?”, it did not specify whether this referred to a psychedelic, a dissociative, or any other psychoactive substance such as cocaine, alcohol, or tetrahydrocannabinol (THC). For this reason, "currently-using" participants were not defined as a fourth, mutually exclusive group alongside the three groups for whom psychedelic-use history could be established (non-users, lifetime users, and recent users), as the specific substance influencing participants at the time of study participation was unknown.

Prior to data collection, the plan had been to divide participants into those using serotonergic psychedelics, glutamatergic agents, or other agents, with the intention of comparing them to a control group of non-users. This stratification sought to differentiate the cognitive effects of serotonergic versus glutamatergic agents, given the distinct expression patterns of their main target receptors in the brain. However, during data collection, it became evident that the number of participants who exclusively used glutamatergic substances such as ketamine or PCP was too small to allow statistical analysis. This may have been due to the strong dissociative effects of ketamine, which make it less likely to serve as an initial substance of use or because our sample predominantly comprised US citizens, where ketamine use is less prevalent (55).

All statistical tests were performed to differentiate between the acute, subacute, and long-term effects of psychedelics on task performance and mental health outcomes. To evaluate differences between the main outcome variables (accuracy and reaction time for each cognitive task), the Kruskal-Wallis test was employed, as data were skewed. *Post-hoc* Dunn’s tests were applied for significantly different variables between the three groups. Because data were not normally distributed, a correlation between cognitive outcomes and drug use was calculated with partial Spearman’s correlations, with age at first use, sex, education level, age, use of non-psychedelics, and mental health diagnoses as covariates. Spearman correlations were further used to test for correlations between cognitive outcomes and average drug dose, with sex, education level, age, and use of non-psychedelics as covariates. In an exploratory analysis, the covariate “currently-using” was added to address the bias of participants among both user groups stating that they were under the influence of a psychoactive drug during study participation. Mann-Whitney-U-tests were used to test for differences in cognitive outcomes and mental health outcomes between the “currently-using” group and users not under the influence of a psychoactive drug during the tasks. To test for differences in the mental health questionnaires, the Kruskal-Wallis test followed by Dunn’s tests were applied. Bonferroni correction was used to correct for multiple testing for *post-hoc* Dunn’s test and for Mann-Whitney-U-tests. Dose-dependent task outcomes were tested via Spearman correlations for the most used drugs (LSD, psilocybin, alcohol, and THC).

## 3 Results

### 3.1 Participants and demographic data

Of the 1572 participants who clicked through the study link, 812 were excluded for exceeding the time limit of 40 minutes (see Supplement for details regarding the number of excluded participant trials for the cognitive tasks). Most excluded participants (70%) were excluded during the first phase of the experiment, before beginning any of the tasks. The included participants took, on average, 24 minutes to complete the experiment, suggesting that most excluded participants likely aborted the experiment rather than running out of time. One participant was excluded because they were underage. Of the 759 participants included in the study, 18.97% (n=144) were in the non-user group, 23.84% (n=181) were in the lifetime user group (meaning they had ingested at least one substance in their lifetimes), and 57.18% (n=434) were recent users (defined as participants who had ingested at least one substance in the week prior to study participation).

In the two user groups, 39.66% (n=301) participants stated that they were under the influence of a psychoactive drug while completing the study. Within this “currently-using” subsample, 7.64% (n=23) participants stated that they had not taken a psychedelic drug in the week prior to the study and were hence counted as lifetime users. To address this confounding factor while still allowing for an analysis with time-dependent outcomes, an additional exploratory analysis was conducted that excluded the 23 “currently-using” participants, enabling us to better distinguish time-dependent drug effects. An additional exploratory analysis was conducted that excluded all 301 “currently-using” participants to address the bias of possible acute effects. No fourth, mutually exclusive group of "currently using" participants was created, as it remained unclear which substance they were under the influence of during study participation. The questionnaire item only asked whether respondents were “currently under the influence of a psychoactive substance”, which could include THC, cocaine, alcohol, or psychoactive substances like caffeine that were not assessed in the study. Eight participants (1.05%) who stated that they had never used a psychedelic but then listed use of at least one psychedelic substance were reclassified as non-users (an additional exploratory analysis excluding these participants did not show diverging statistical outcomes).

Demographic data showed that the sample was mostly male (73%), in early adulthood (32.62 ± 5.93 years), well-educated (80.1% of participants had a tertiary education degree), and from Western countries; 71.5% were US citizens. The most common self-reported psychiatric condition among all participants was depression (31.5%), followed by self-reported addiction (30.3%) and no mental health condition (“None”) (26.2%) (**Figure 1**; **Supplementary Table S1**).

**Figure 1:**
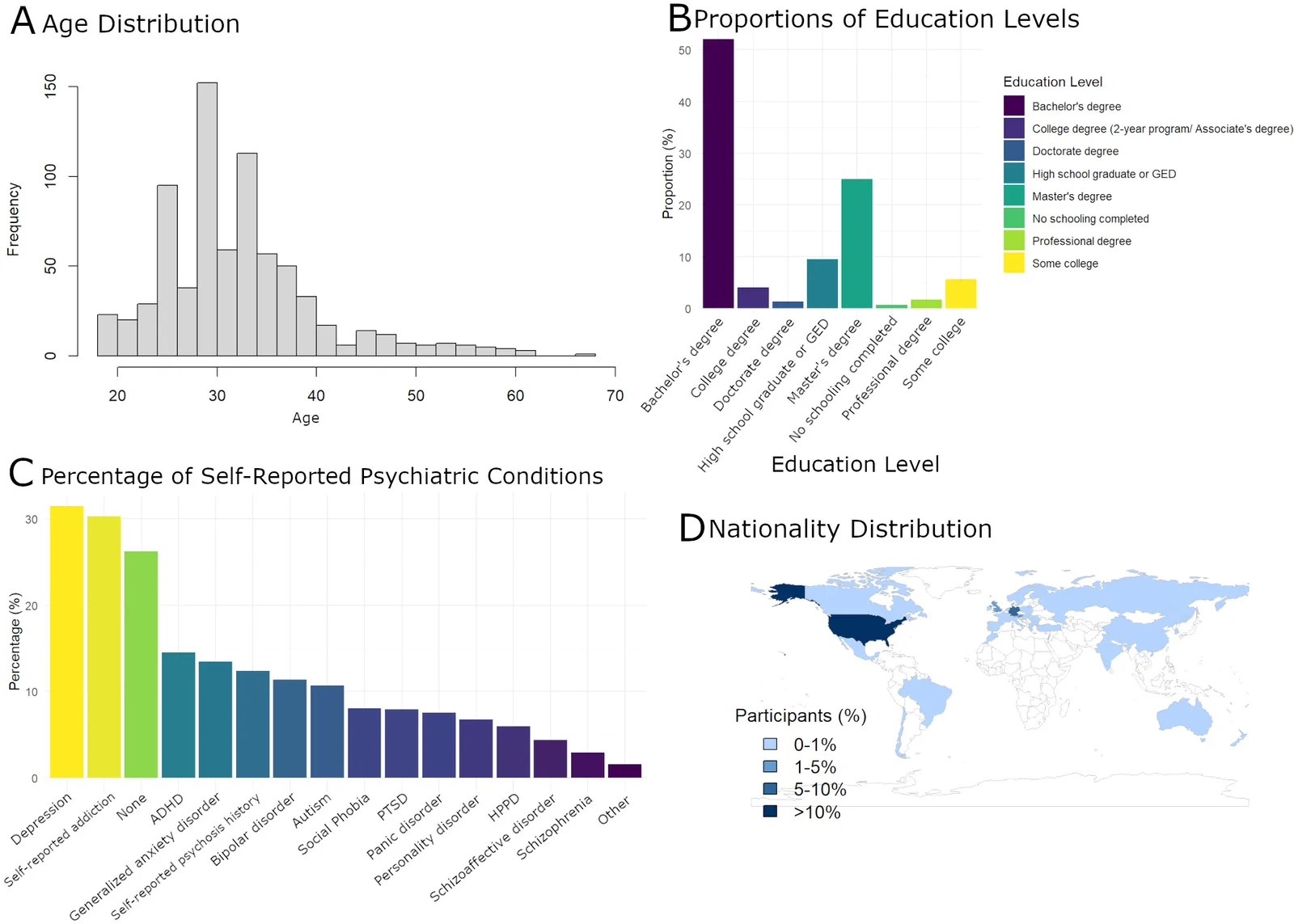
Participant demographics (n = 759). **(A)** The age distribution of participants. **(B)** Participants’ education levels. **(C)** Percentage of participants’ self-reported psychiatric conditions. **(D)** World map showing nationality distribution of participants. Darker shades of blue indicate higher percentages of participants originating from a country.

### 3.2 Drug and alcohol use

With regard to lifetime consumption, the most commonly used drug was alcohol (71.0%), with a median amount of 130.5 standard drinks per month (a standard drink is approximately 33cl or 12oz of beer at 5% alcohol by volume/15cl or 5oz of wine at 12% alcohol by volume/4cl or 1.5oz of spirits at 40% alcohol by volume (a “shot”), or approximately 14g of pure ethanol (interquartile range (IQR) = 407.75 standard drinks). The second most frequently used substance was psilocybin (“magic mushrooms”) (47.0%); the median dose was 18.0g (IQR = 48.0g) of dried mushrooms, the median number of lifetime uses was 126.5 (IQR = 434.75), and the median amount of times used in the week prior to study participation (recent frequency) was 26.5 (IQR = 51.25). The third most frequently used substance was LSD (46.1%); the median dose was 125.0mcg (IQR = 166.75mcg), the median number of lifetime uses was 133.0 (IQR = 489.5), and the median recent frequency was 23.0 (IQR = 49.0) (**Figure 2**; **Supplementary Table S2**). These were followed by, in order of frequency, THC (41.8%, median dose of 25.0g per month (IQR = 27.12g)), cocaine (40.1%, median dose of 34.5g per month (IQR = 26.19g)), MDMA (24.9%, median dose of 100.0mg per month (IQR = 80.0mg), and opioids (23.6%, median dose of 38.0g per month (IQR = 25.95g)).

**Figure 2:**
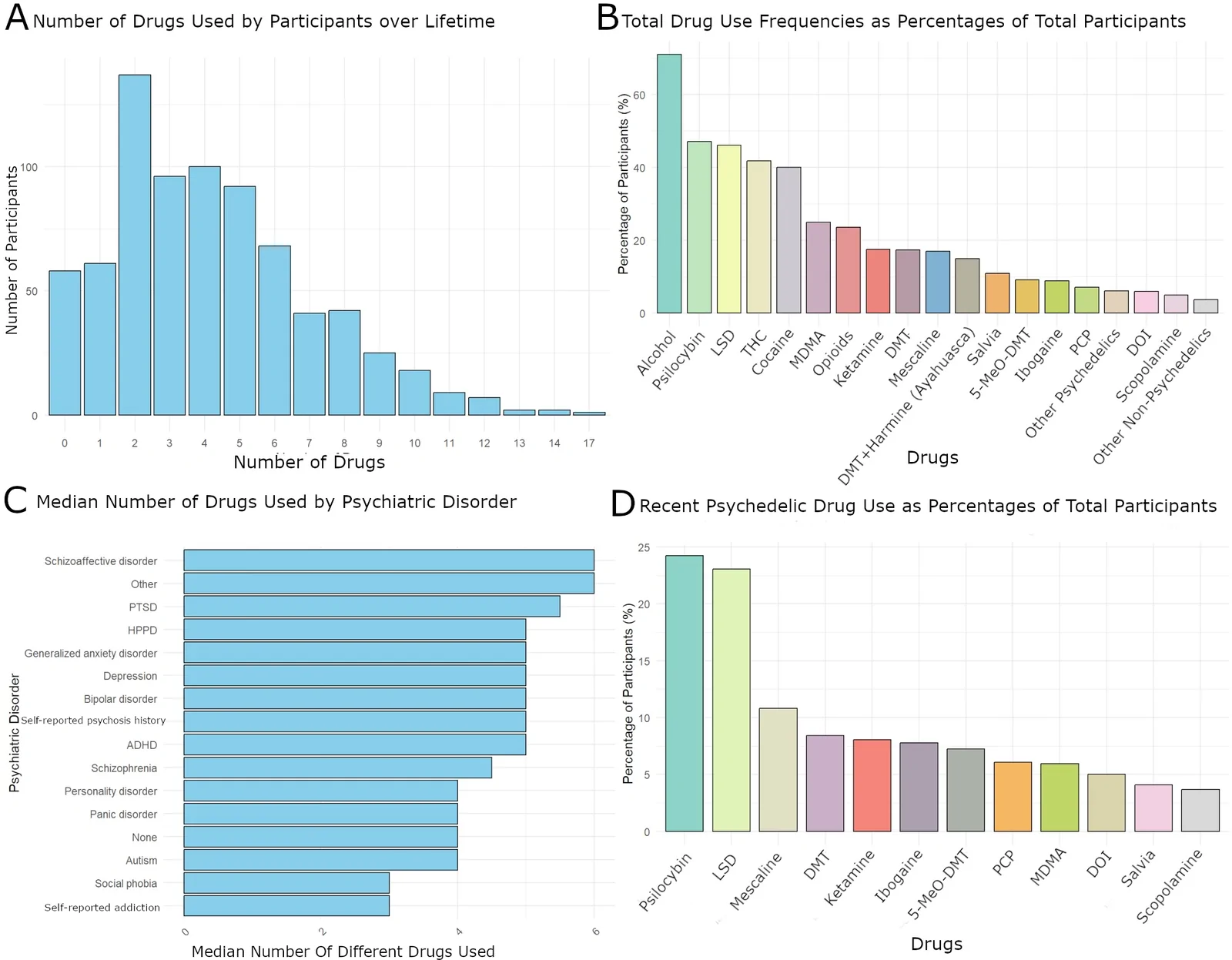
Psychedelics and additional drug use. **(A)** The number of different drugs (psychedelic and other drugs) that participants (n=759) reported using over their lifetime. **(B)** The percentage of drug use frequencies of participants. DMT + Harmine (Ayahuasca) refers to Ayahuasca; in the rest of the manuscript/supplement the term “DMT” also refers to ayahuasca. **(C)** The median number of different drugs used per participant grouped by self-reported psychiatric conditions. Participants with schizoaffective disorder and post-traumatic stress disorder (PTSD) reported the highest median number of different substances. **(D)** Psychedelic drug use and use of substances with hallucinogenic properties in the week prior to study participation (recent psychedelic drug use). 5-MeO-DMT, 5-methoxy-N,N-dimethyltryptamine; DMT, N,N-dimethyltryptamine; DOI, 2,5-dimethoxy-4-iodoamphetamine; LSD, lysergic acid diethylamide; MDMA, 3,4-methylenedioxymethamphetamine; PCP, phencyclidine; THC, tetrahydrocannabinol.

In this study, 17.5% of respondents reported lifetime ketamine use (median dose of 66.0mg, IQR = 80.0mg, median number of lifetime uses of 240.0 (IQR = 454.5), and median recent frequency of 27.0 (IQR = 30.0)). Other serotonergic psychedelics, such as DMT (“ayahuasca”), mescaline, and ibogaine were used less frequently (for dosages and frequencies, see **Supplementary Table S2**). Most (92.36%) participants reported having used several different substances across their lifetimes, with two (18.05%), three (12.65%), and four (13.18%) substances being the next most commonly reported, indicating a prevalent pattern of polydrug use in this sample.

In the week prior to taking part in the study, the most commonly used psychedelic drugs were psilocybin (23.8%) and LSD (22.5%), followed by mescaline (10.6%) and DMT (8.2%), showing a strong preference for serotonergic psychedelics over substances like ketamine (7.5%) or MDMA (5.5%) (**Figure 2**).

The most common setting reported for a psychedelic experience was recreational psychedelic drug use (n=300, and n=216 with friends), followed by psychedelic drug use in the context of research (n=192) and in therapy (n=173). The mean age for first-time psychedelic use was 20.5 years (± 8.15 years) (**Supplementary Figure S1**).

### 3.3 Neurocognitive tasks

Five cognitive tasks were used to measure the three cognitive domains of working memory, selective attention, and spatial/visual perception: the digit span text entry task, the 2-back task, the dot-probe task, the visual search task, and the 2D mental rotation task. The main outcome variables included number of correctly answered trials (accuracy) and the mean reaction time for each cognitive task, meaning that there were 10 variables of interest. Significant differences were observed between the non-user, lifetime user, and recent user groups for most of the 10 main outcome variables (**Table 1**; **Figure 3**; for descriptive outcomes see **Supplementary Table S3**).

**Table 1:** Kruskal-Wallis test for five cognitive tasks and mental health questionnaires for all participants (left), and Mann-Whitney-U Test for five cognitive tasks between “currently using” users and users who were not under the influence of a psychoactive drug during study participation (right).

Dependent variable	Test statistic	*P*-value
Reaction time dot-probe	111.11	<0.0001
Accuracy dot-probe	129.94	<0.0001
Reaction time 2D-rotation	100.04	<0.0001
Accuracy 2D-rotation	161.19	<0.0001
Reaction time visual Search	0.36	0.8348
Accuracy visual search	75.82	<0.0001
Reaction time 2-back	3.99	0.1364
Accuracy 2-back	138.53	<0.0001
Reaction time digit span	2.54	0.2812
Accuracy digit span	34.15	<0.0001
PHQ-9	197.91	<0.0001
HPPD	255.61	<0.0001
WHOQOL–BREF-overall	24.64	<0.0001
Physical domain	36.51	<0.0001
Psychological domain	18.042	0.0001
Social domain	55.96	<0.0001
Environmental domain	0.74	0.6927
DES-B	239.06	<0.0001

Left: Results of Kruskal-Wallis Test for differences across the three groups (n=759)—non-users, lifetime users, and recent users—for accuracy (number of correct answers) and mean reaction time for the five cognitive tasks and four mental health questionnaires. PHQ-9, nine-item Patient Health Questionnaire; HPPD, Hallucinogen Persisting Perception Disorder; DES-B, Brief Dissociative Experiences Scale; WHOQOL-BREF, World Health Organization - Quality of Life questionnaire. The WHOQOL-BREF questionnaire is divided into five parts: overall quality of life and physical, psychological, social, and environmental domains. Right: Results of Mann-Whitney-U Test testing for differences for accuracy and mean reaction time between users who were under the influence of a psychoactive drug during task performance and users who were not. Bonferroni correction was applied for multiple testing.

**Figure 3:**
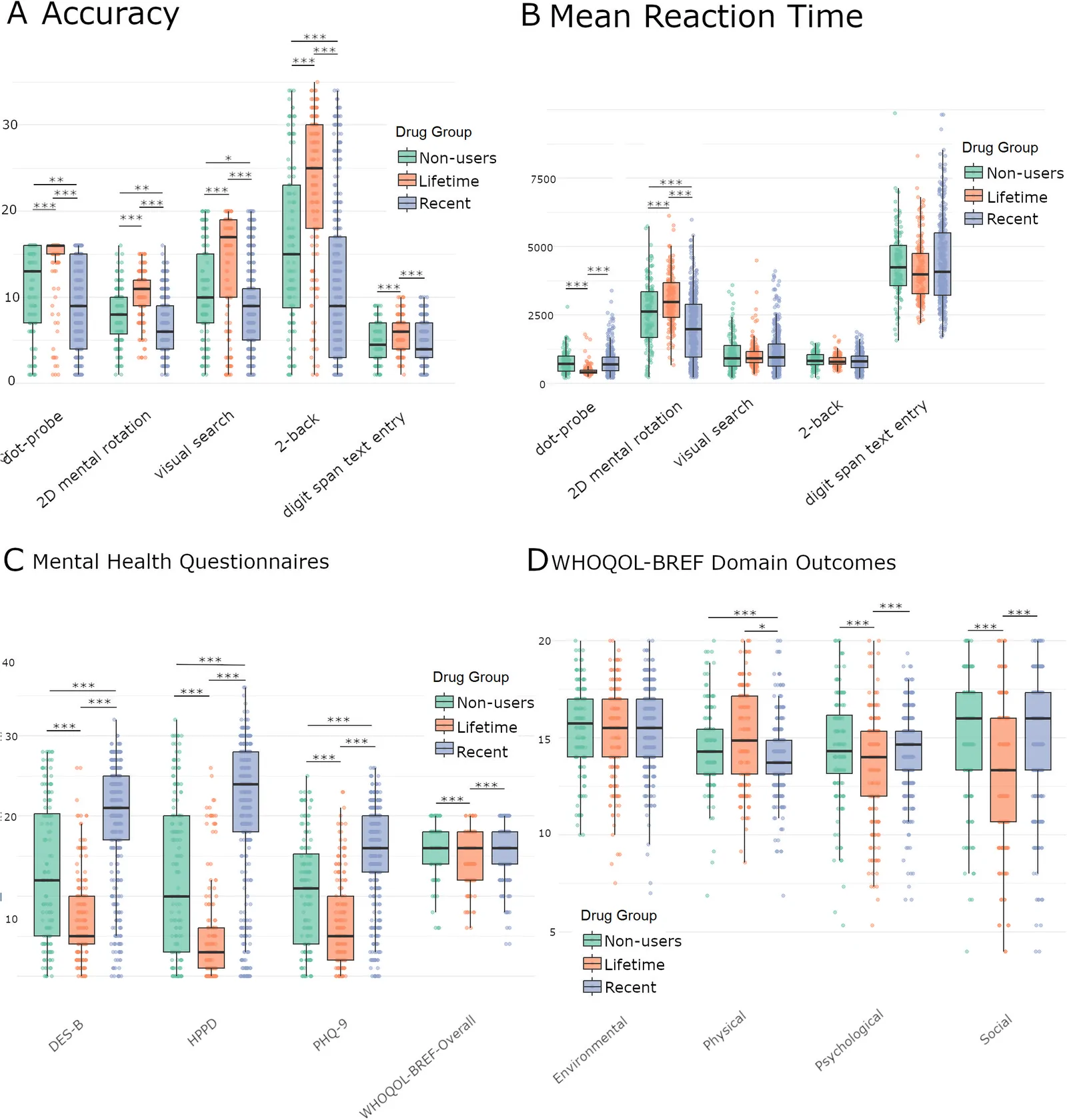
Kruskal-Wallis test of cognitive task performance and mental health by drug use group. Boxplots depicting differences across three drug use groups (n=759) [non-users (green), lifetime users (orange), recent users (blue)] for cognitive performance (accuracy and mean reaction time) and mental health questionnaires. Group differences were assessed using Kruskal-Wallis tests with Dunn’s *post-hoc* comparisons. Significance is indicated as follows: *p < 0.05, **p < 0.001, ***p < 0.0001. **(A)** Accuracy of cognitive task performance across five tasks: dot-probe, 2D mental rotation, visual search, 2-back, and digit span text entry. **(B)** Mean reaction time for each cognitive task listed in **(A)**. **(C)** Outcomes from mental health questionnaires, including the Brief Dissociative Experiences Scale (DES-B), Hallucinogen Persisting Perception Disorder scale (HPPD), nine-item Patient Health Questionnaire (PHQ-9), and the overall score from the World Health Organization Quality of Life Questionnaire (WHOQOL-BREF). **(D)** WHOQOL-BREF domain-specific outcomes: environmental, physical, psychological, and social.

In the digit span text entry task, which assesses working memory, lifetime users were significantly more accurate than either non-users (Z=-4.065, p<0.0001) or recent users (Z = 5.703, p<0.0001), while there was no significant difference between non-users and recent users (Z = 0.392, p=0.6954). Reaction times did not differ significantly between the three groups (χ^2^(2)=2.537, p=0.281).

In the 2-back task, which also assesses working memory, reaction times did not differ significantly between the groups (χ^2^(2)=3.985, p=0.136), but accuracy was significantly higher in lifetime users versus non-users (Z=-5.578, p<0.0001) or recent users (Z = 11.74, p<0.0001), and recent users performed significantly worse than non-users (Z = 4.112, p=0.0001).

Dunn’s *post-hoc* analysis revealed significant group differences in the dot-probe task, which measures attentional bias, with lifetime users performing significantly more accurately than either non-users (Z=-5.896, p<0.0001) or recent users (Z = 11.397, p<0.0001). In contrast, recent users performed significantly worse than non-users (Z = 3.509, p=0.0005). Lifetime users showed significantly shorter reaction times than non-users (Z = 8.308, p<0.0001) and recent users (Z=-9.927, p<0.0001), while no significant difference was found between non-users and recent users (Z = 0.541, p=0.5889).

In the visual search task, which measures spatial and visual perception, no significant differences were observed in reaction time (χ^2^(2)=0.361, p=0.835). Lifetime users demonstrated significantly higher accuracy than either non-users (Z=-4.547, p<0.0001) or recent users (Z = 8.701, p<0.0001). Recent users again performed significantly worse than non-users (Z = 2.741, p=0.0042).

Finally, in the 2D mental rotation task—which also evaluates spatial and visual perception—lifetime users had significantly higher accuracy than either non-users (Z=-7.152, p<0.0001) or recent users (Z = 12.696, p<0.0001), and recent users showed significantly lower accuracy than non-users (Z = 3.379, p=0.0007). With regard to reaction time in the 2D mental rotation task, lifetime users had significantly longer reaction times than non-users (Z=-3.749, p=0.0002) or recent users (Z = 9.784, p<0.0001), and recent users had significantly shorter reaction times than non-users (Z = 4.651, p<0.0001).

Partial correlation testing between task accuracy and recent drug use revealed a significant correlation for all five cognitive tasks (dot-probe task: r=−0.304, p<0.001; 2D mental rotation task: r=−0.332, p<0.001; visual search task: r=−0.161, p<0.001; 2-back task: r=−0.305, p<0.001; digit span text entry task: r=−0.097, p=0.0424). Reaction time outcomes were significantly positively correlated with recent psychedelic drug use for the dot-probe task (r=0.258, p<0.001) and negatively with the 2D mental rotation task (r=−0.232, p<0.001). With regard to the correlation between lifetime use and task accuracy, a significant negative correlation was revealed for the dot-probe task (r=-0.154, p<0.001), the 2D mental rotation task (r=-0.160, p<0.001), the visual search task (r=-0.096, p=0.0436), and the 2-back task (r=-0.223, p<0.001). Mean reaction time was negatively correlated for the 2D mental rotation task (r=-0.187, p<0.001) (**Supplementary Table S4**; **Supplementary Figure S2**). When exploratory correlation testing was conducted with “currently-using” as an additional covariate, the same outcomes remained statistically significant, although with smaller effect sizes (see Supplement).

Corrected Spearman correlations showed dose-dependent effects for LSD, psilocybin, alcohol, and THC for accuracy across all five cognitive tasks, and higher median doses correlated with lower accuracy (r=-0.551 to -0.106; p=0.0137 to p<0.0001). Speed-related correlations showed varying outcomes (**Supplementary Table S5**). However, a corrected Spearman correlation for drug dose when tested with the covariates of sex, education level, age, and non-hallucinogen use showed no significant association.

To assess the impact of acute psychoactive effects, a Mann-Whitney U-test compared participants who self-reported active psychoactive intoxication during task performance with those who did not. For the 301 participants stating acute psychoactive intoxication, 23 (7.64%) belonged to the lifetime user group and 278 (92.36%) to the recent user group. The first group exhibited significantly lower accuracy across all five cognitive tasks (**Table 1**; **Figure 4**). In the exploratory analysis that excluded all “currently-using” participants (n=301), the recent user group no longer showed comparably poor performance in accuracy and appeared more similar to the lifetime user group. Reaction time outcomes did not differ (**Supplementary Tables S9**, **S10**).

**Figure 4:**
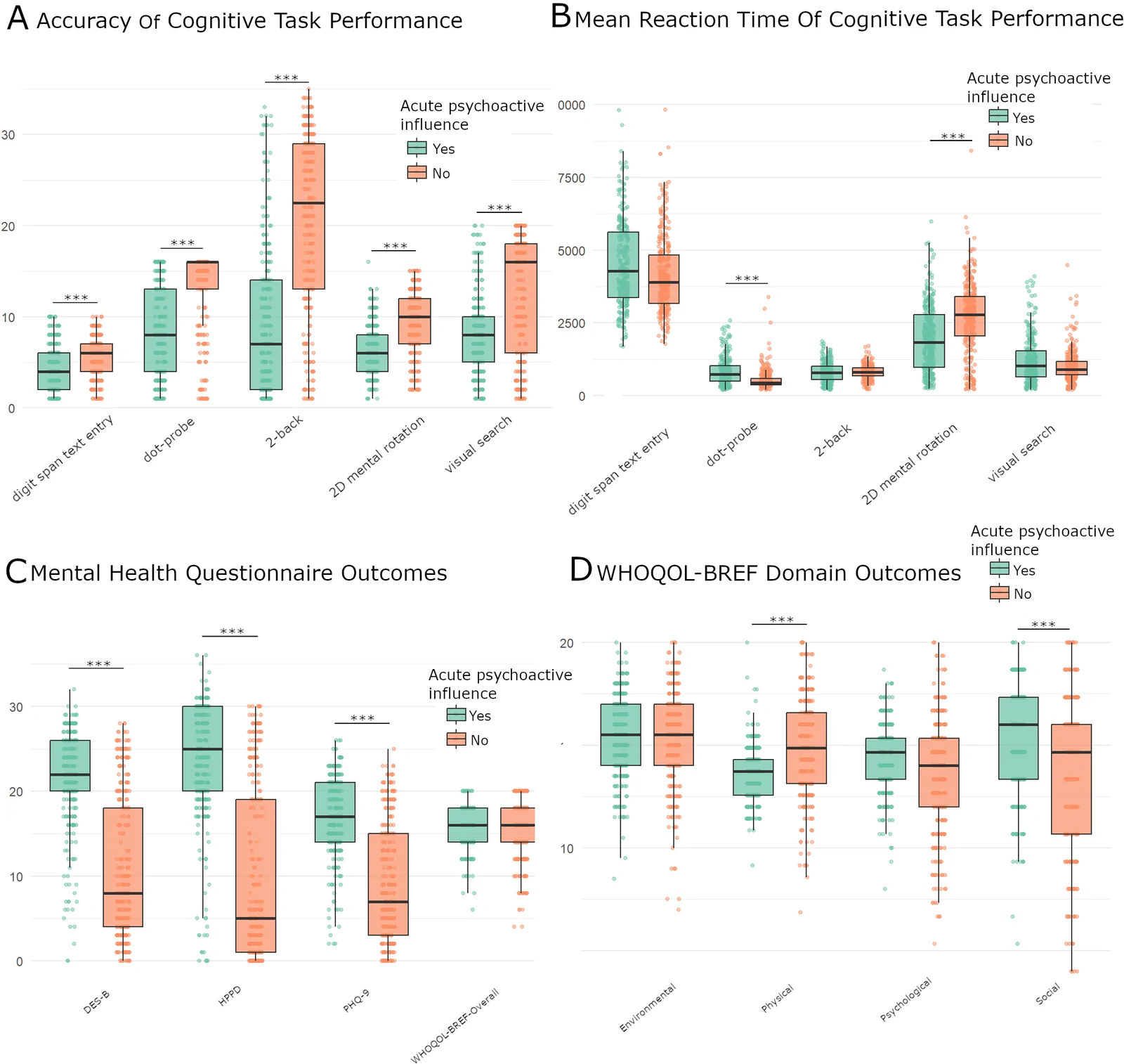
Mann-Whitney U-test of cognitive and mental health outcomes grouped by acute psychoactive drug influence. Boxplots depicting differences between two groups of psychoactive drug users (1=participants who reported that they were under the influence of a psychoactive drug during task performance (green, n=301), 2=participants who stated that they were NOT under the influence of a psychoactive substance during task performance [orange, n=314)]. Statistical comparison between the groups as performed using the Mann–Whitney U test, with significance levels indicated as follows: *** =* p *< 0.0001. **(A)** Accuracy and **(B)** mean reaction time of cognitive task performance across five domains: dot-probe, 2D-mental-rotation, visual search, 2-back, and digit span text entry. **(C)** Mental health outcomes assessed with the Brief Dissociative Experiences Scale (DES-B), Hallucinogen Persisting Perception Disorder (HPPD) score, and the nine-item Patient Health Questionnaire (PHQ-9). **(D)** Self-reported quality of life outcomes measured with the WHOQOL-BREF (World Health Organization-Quality of Life) questionnaire across four domains (environmental, physical, psychological, social) as well as Overall score **(C)**.

### 3.4 Mental health

Group comparisons revealed significant differences in depressive symptoms (PHQ-9: χ^2^(2)=197.9, *p* < 0.0001), HPPD scores (χ^2^(2)=255.6, *p* < 0.0001), and dissociative symptoms (DES-B: χ^2^(2)=239.059, *p* < 0.0001). Across questionnaires, lifetime users showed significantly better mental health outcomes than recent users (**Figure 4**; descriptive outcomes appear in **Supplementary Table S6**). In addition, *post-hoc* pairwise comparisons showed that lifetime users had significantly lower depressive symptom scores than both non-users (*Z* = 4.376, *p* < 0.0001) or recent users (*Z*=–13.5, *p* < 0.0001) and that recent users reported significantly higher depression scores than non-users (*Z*=–7.369, *p* < 0.0001). For HPPD, lifetime users scored significantly lower than either non-users (*Z* = 5.563, *p* < 0.001) or recent users (*Z* = -15.5, *p* < 0.0001), with recent users again scoring higher than non-users (*Z* = -7.8, *p* < 0.0001). Similarly, dissociative symptom scores were lowest in lifetime users, who differed significantly from both non-users (*Z* = 5.591, *p* < 0.0001) and recent users (*Z* = -15.077, *p* < 0.0001), while recent users again showed significantly higher dissociative symptoms than non-users (*Z* = -7.380, *p* < 0.0001).

The WHOQOL-BREF questionnaire is divided into five different domains: physical, psychological, social, environmental, and overall quality of life. Significant differences were found in the physical (*χ*^2^(2)=36.5, *p* < 0.0001), social (*χ*^2^(2)=55.959, *p* < 0.0001), and psychological domains (*χ*^2^(2)=18.0, *p* = 0.0001), and in overall quality of life (*χ*^2^(2)=24.642, *p* < 0.0001), but no significant difference was observed in the environmental domain (*χ*^2^(2)=0.7, *p* = 0.6927) (**Figure 3**). *Post-hoc* pairwise comparisons showed that lifetime users had significantly lower scores in the overall quality of life domain than either non-users (*Z* = 4.381, *p* < 0.0001) or recent users (*Z* = -4.386, *p* < 0.0001), while no difference was observed between non-users and recent users (*Z* = 1.052, *p* = 0.2930). In the psychological and social domains, lifetime users again scored significantly lower than either non-users (psychological: *Z* = 3.615, *p* = 0.0003; social: *Z* = 6.049, *p* < 0.0001) or recent users (psychological: *Z* = -3.870, *p* = 0.0001; social: *Z* = -7.031, *p* < 0.0001), with no significant differences observed between non-users and recent users (psychological: *Z* = 0.637, *p* = 0.5242; social: *Z* = 0.555, *p* = 0.5791). In the physical domain, recent users had significantly lower scores than either non-users (*Z* = 2.761, *p* = 0.0058) or lifetime users (*Z* = 5.922, *p* < 0.0001); the difference between non-users and lifetime users was not significant (*Z* = -2.314, *p* = 0.0207).

Notably, participants who reported being under the influence of a psychoactive drug during task performance showed significantly higher depressive symptom scores (PHQ-9; *U* = 76727.0, *p* < 0.0001), HPPD scores (*U* = 79907.0, *p* < 0.0001), and dissociative symptom scores (DES-B; *U* = 78778.0, *p* < 0.0001) than users who were not acutely intoxicated. They also exhibited higher scores in the physical domain (*U* = 31084.0, *p* < 0.0001) of the WHOQOL-BREF. In contrast, they scored significantly lower in the social domain of the WHOQOL-BREF (*U* = 59415.0, *p* < 0.0001) compared to users who were not in an acute state (**Figure 4**).

In the exploratory analysis excluding the 301 “currently-using” participants, recent users still showed the lowest outcomes on average, but the differences compared with lifetime users were no longer statistically significant, primarily due to the wider confidence intervals resulting from the reduced sample size (**Supplementary Tables S9**, **S10**).

## 4 Discussion

This cross-sectional, international study used online tasks to investigate the acute, subacute, and long-term effects of psychedelic drug use on neurocognitive function and mental health outcomes. By comparing non-users, lifetime users, and recent users, the study sought to disentangle the temporally specific impact of psychedelic and additional drug use on cognitive and psychological domains. Our study used objective tasks to provide new insights into the potential cognitive impacts of classic serotonergic psychedelics and ketamine, identifying dose- and time-dependent effects on selective attention, working memory, and visual/spatial perception.

Five cognitive tasks were used to assess the three cognitive domains of interest: working memory, selective attention, and spatial/visual perception. These included the digit span text entry and 2-back tasks for working memory, the dot-probe task for selective attention, and the visual search and 2D mental rotation tasks for spatial/visual perception. Significant differences were observed between non-users, lifetime users, and recent users across all five cognitive task outcomes. Broadly, recent users displayed significantly lower accuracy than non-users and lifetime users, suggesting potential (sub)acute cognitive impairments across the three cognitive domains.

With regard to working memory, these findings support prior research. For instance, one double-blind, placebo-controlled study found that working memory was impaired after high doses (250mcg/kg) of psilocybin (56), though the sample size was small (n=12) and a different task (spatial span task) was used to measure working memory. Another double-blind, placebo-controlled study (n=20) also used the 2-back task and found that psilocybin increased reaction time but did not affect accuracy (57). In the present study, lifetime users significantly outperformed non-users and recent users on both tasks that measured working memory (2-back and digit span text entry), though no differences in reaction time were observed. However, given the notable limitations and confounding factors enumerated below, it cannot be concluded that lifetime use might potentially enhance memory capacity. Furthermore, accuracy was negatively correlated with drug use in both lifetime and recent users.

With regard to selective attention, our findings of (sub)acute impairment in recent users and increased accuracy in lifetime users are in line with previous research, though data on attentional bias are scarce (27). Most notably, 39.7% (n=301) of the participants, all of whom belonged to the lifetime or recent user groups, stated that they were under the influence of a psychoactive drug while taking part in the study, preventing us from differentiating between acute and subacute effects. Impairment might thus appear as an acute effect, given that prior studies observed no subacute impairments in attention. In an exploratory analysis that excluded participants stating that they were under the influence of a psychoactive drug during the study, recent users no longer performed significantly worse than non-users. One open-label study of 24 participants with MDD found no impairment in selective attention one and four weeks after administration of one moderate dose (20mg/70 kg) and one high dose (30mg/70 kg) of psilocybin (58). Another double-blind, placebo-controlled study found no subacute impairment of attention 24 hours after LSD administration (50mcg) (59). However, most research has examined attentional bias in the context of microdosing, and those studies obtained mixed results ranging from reports of no observable effects (32) to enhanced attentional performance (30). These inconsistencies highlight the methodological challenges associated with comparing studies that lack standardization, particularly concerning varying doses, sample sizes, and cognitive tasks (16). Further research investigating other domains of cognition show equally varying outcomes. For example, one study found improved cognitive flexibility after two macrodose psilocybin administrations (20mg/70 kg and 30mg/70 kg, respectively), whereas a double-blind, placebo-controlled study noted acutely impaired creative thinking after a macrodose of psilocybin (0.17mg/kg), followed by increased creative thinking seven days later (60).

When considering dose-dependent effects, no significant associations were noted between dose and cognitive task outcomes after controlling for covariates. However, analyses focusing on individual substances—LSD, psilocybin, alcohol, and THC—consistently found that higher doses were associated with impairments across all three cognitive domains. This is in line with previous research suggesting that high doses impair cognitive functioning, while lower doses may facilitate enhancement (15, 27). Nevertheless, longitudinal and more consistent study designs are needed before conclusions can be drawn beyond acute effects only (16).

Interestingly, lifetime users demonstrated higher accuracy than non-users or recent users across all five cognitive tasks. However, these findings were not supported during correlation testing, suggesting no potential long-term cognitive benefits associated with psychedelic experiences, and paralleling previous research that found that psychedelics had no long-term effects on cognition (15, 61). In addition, because participants who reported being under the influence of a psychoactive drug during the cognitive task were included in the lifetime users group, it is difficult to disentangle acute from long-term effects. To address this bias, an exploratory statistical analysis was conducted after excluding the 23 “currently-using” lifetime user group participants (**Supplementary Tables S7**, **S8**). The outcomes of the Kruskal-Wallis test and the *post-hoc* Dunn’s test remained unchanged, as did the results of the correlation analyses. However, after excluding all “currently-using” participants, recent users no longer had significantly lower accuracy than non-users across all five cognitive tasks, suggesting that the initially observed impairment among recent users was primarily driven by acutely intoxicated individuals and likely reflects acute rather than subacute effects. As the type of psychoactive substance used at the time of study participation was not assessed, it remains unclear which drug class contributed to these effects. Future research should include systematic substance assessments.

It is also interesting to note that recent users in this analysis had significantly higher depressive symptom scores (as assessed by the PHQ-9) and a higher prevalence of HPPD symptoms and dissociative symptoms (as assessed by the DES-B) than either non-users or lifetime users, possibly reflecting that acute or subacute after-effects might cause psychological distress. The fact that recent users did not perform significantly worse than non-users after the “currently-using” subsample was excluded from the analysis suggests that observed impairments were mostly driven by participants under acute intoxication during study participation. It is also important to take the bilateral relationship between mental health and psychedelic substance use into account, as participants with mental health conditions might also have turned to psychedelic experiences in an attempt at self-medication or in a therapeutic or research context. It should also be noted that the prevalence of depressive disorders in our sample was much higher (31.5%) than typically observed in a population study; for instance, one US study reported a prevalence of 18.4% in 2020 (62). Interestingly, lifetime users in this study had significantly lower depressive symptom scores, fewer dissociative symptoms, and lower HPPD scores than either recent users or non-users. While previous research found no negative long-term mental health outcomes in psychedelic drug users (11, 63), it is not possible to draw the same conclusion from our results, as recent users who showed higher scores might also be lifetime users.

It should also be noted that on the WHOQOL-BREF, lifetime users scored lower in the psychological and social domains than non-users or recent users, potentially suggesting that psychedelic use has long-term social effects. These outcomes might be due to difficulties integrating challenging psychedelic experiences or altered social behavior. However, the finding that lifetime users reported fewer depressive symptoms but lower psychological and social QOL- scores than other user groups might also reflect that symptom questionnaires and broader questionnaires representing more complex well-being constructs capture different approaches to mental health. However, these findings may also be influenced by selection bias, as individuals with a strong interest in psychedelics or particularly positive or negative experiences with these substances may have been more likely to participate in the study.

Despite the strengths of the study—which include its international, naturalistic design and easy accessibility—several limitations warrant attention. First, in an online study, there is little control over the setting in which participants complete questionnaires and tasks, and considerable variability may exist with regard to the type of devices used or other environmental distractions, leading to variability in the dataset.

Second, potential biases might be present when self-reporting drug use and mental health symptoms, either as a result of difficulties in accurately recalling substance or dose or because of potential social pressure. As a salient example, the psilocybin content of specific species of psilocybin-containing mushrooms often remain unknown to users; psilocybe cubensis, the most commonly used species, contains approximately 10 mg of psilocybin per gram of dried mushroom (64), which served as the reference dose in this study.

Third, while there is evidence that Amazon Mechanical Turk participants show comparable attentiveness and similar results in cognitive tasks as offline participants (65, 66), in an online study it is not possible to evaluate the accuracy or honesty of participant responses; for example, the reported average dose of psychedelic drugs such as dried mushrooms, expressed in grams, greatly exceeded typical doses. Fourth, this study did not take into account nicotine use or prescribed medications, including those with neurocognitive (side) effects.

Fifth, a major limitation of the present study is that some recent and lifetime users reported being under the influence during participation, substantially limiting our ability to distinguish acute from subacute effects. The observed cognitive impairments may therefore have been driven primarily by acute psychoactive substance effects in the currently using subgroup rather than by recent psychedelic exposure itself. Relatedly, the psychoactive substances that participants reported being under the influence of were not specified, making it impossible to determine whether the observed acute effects were attributable to psychedelics versus alcohol or other substances. Although this bias was addressed by conducting an exploratory additional analysis that excluded the “currently-using” participants from the lifetime user group as well as another analysis that excluded all “currently-using” participants, which allowed us to differentiate between long-term and (sub)acute effects, these analyses only partially migitated the limitation and cannot fully disentangle temporal effects. Future work should consider incorporating drug level assessments at screening and during task performance, ideally within controlled settings, to reduce the risk of acute substance influence.

Sixth, the arbitrary nature of our group configuration—for example, participants who used psychedelic drugs seven days before study participation were counted as recent users while those who had used psychedelic drugs eight days beforehand were counted as lifetime users—limited the real-world generalizability of the results.

Seventh, the total number of substances used was not reflected in the outcomes of the Kruskal-Wallis test, thus not taking into account that recent users were most likely the subgroup with the heaviest drug use history. Furthermore, most participants stated polydrug use, leading to potential confounding effects.

Eighth, like most psychedelic research, a selection bias may exist regarding the prevalence of psychedelic drug use in association with socioeconomic and sex differences. Data from the Global Drug Survey suggest that the prevalence of lifetime psychedelic drug use is 17% between the ages of 21 and 64 (67), with 16.1% reporting psilocybin use in the past 12 months (68), revealing much lower prevalence rates than in our study (47% of participants reporting lifetime psilocybin use and 46.1% of participants LSD lifetimes use, respectively). This difference might stem from our recruitment strategies, which specifically targeted participants interested in psychedelic research. Recruitment strategies might have also skewed data concerning age.

Ninth, there is a potential selection bias in that chronic psychedelic users are likely those who have not experienced adverse effects, as individuals with negative initial experiences are less likely to continue use. However, our broad definition of lifetime users (at least one lifetime use but no recent use of a psychedelic substance) resulted in a heterogeneous group, which limits the adequate interpretability of long-term effects.

Finally, our sample was homogeneous, well-educated, primarily male, and from predominantly Western countries. While this might not be an accurate reflection of the average psychedelic drug user, most research into psychedelics have observed similar participant samples (15), suggesting that more diverse participant samples and adjusted recruitment strategies will be crucial for establishing a safety profile for these agents across the general population. To address some of these limitations, our analyses used validated cognitive tasks and included covariates such as psychiatric condition, age at first use, age, sex, use of non-hallucinogens, and education level.

Despite these limitations, this large-scale, international, naturalistic study offers novel insights into the cognitive and psychological correlates of psychedelic use by distinguishing between non-users, recent users, and lifetime users across multiple cognitive domains. Our findings provide evidence of (sub)acute cognitive impairments in the group of recent users in the three cognitive domains of working memory, selective attention, and visual/spatial perception; specifically, recent use was associated with reduced task accuracy. Although lifetime users demonstrated superior performance in several cognitive tasks, these apparent benefits were not supported by correlation analyses that tested for correlations between the total amount of psychedelics and dissociative agents used and cognitive task outcomes, suggesting that lifetime use does not confer any long-term cognitive advantages. Furthermore, while recent users reported elevated levels of depressive, dissociative, and HPPD symptoms, lifetime users reported fewer symptoms than either of the other groups, aligning with previous findings that psychedelics have no long-term adverse mental health effects. Nevertheless, lower scores on psychological and social quality-of-life domains among lifetime users raise the possibility of subtle, long-term psychosocial consequences, potentially related to the integration of challenging psychedelic experiences. Alternatively, the observed long-term effects may reflect pre-existing psychological vulnerabilities in individuals who turned to psychedelics for help, rather than consequences of psychedelic use itself. Although this study was an exploratory cross-sectional online study that required a more vague interpretation of long-term, subacute, and acute effects, future longitudinal, lab-, and placebo-controlled designs with standardized dosing and diverse, representative samples are needed to deepen our understanding of the complex and dose-dependent cognitive and psychological consequences of psychedelic use.

## Data Availability

The raw data supporting the conclusions of this article will be made available by the authors, without undue reservation.

## Author disclaimer

The contributions of the NIH authors are considered Works of the United States Government. The findings and conclusions presented in this paper are those of the authors and do not necessarily reflect the views of the NIH or the U.S. Department of Health and Human Services.
