# Supplementary material for: Effects of psychedelic drug use on neurocognitive function and the psychological and social domains of quality of life: an international online study
Source: Front Psychiatry. 2026 Sep 24;17:1843186. doi: 10.3389/fpsyt.2026.1843186 (PMC13645748; doi:10.3389/fpsyt.2026.1843186)
Supplement: Supplementary file 1 [file SupplementaryFile1.docx]

*Stadler et al – Effects of Psychedelic Drug Use on Neurocognitive Function and Psychological and Social Quality of Life Domains: An International Online Study*

**Supplement**

**Supplementary Methods**

Demographic data included age, sex, nationality, highest completed education level, years of education, employment status, work hours per week, number of jobs, annual income in US dollars, kind of residence, marital status, number of children currently being cared for, and level of disability.

Five cognitive tasks provided by Gorilla.sc were used to measure working memory, selective attention, and spatial/visual perception.

*N-back task*: In the n-back task, which measures working memory, participants must recall the letter presented n trials earlier. In this version of the task, a 2-back task, participants had to remember the letter shown two trials prior. If no response was given within 2000 ms, the task automatically moved to the next trial, and the trial was considered incorrect. Performance was measured based on the number of correct answers and reaction time. The task consisted of 35 total trials.

*Digit span text entry task:* In the digit span text entry task, which measures working memory, participants were shown a series of digits they had to remember in the correct order. They were then asked to type in the remembered digits. The task included 10 trials, with each trial featuring a maximum of eight digits. Performance was measured based on the number of correct trials and reaction time.

*Dot-probe task*: The dot-probe task measures selective attention[1]. Two pictures were presented simultaneously, and a dot appeared behind one of them. Participants had to identify the picture containing the dot as quickly as possible. The pictures were shown for 200 ms, and if no response was given within 5000 ms, the task automatically moved to the next trial, and the trial was considered incorrect. Performance was evaluated based on accuracy and reaction time. The task included 16 trials.

*2D mental rotation task:* Visual and spatial perception were measured using the 2D mental rotation task[2]. Participants had to identify the two-dimensionally rotated version of a target object. The target object was displayed for a maximum of 1500 ms. Performance was measured by number of correct trials and reaction time. The task consisted of 16 trials. Task performance is associated with activation of the parietal lobe[3].

*Visual search task:* In the visual search task, which measures visual and spatial perception, participants had to locate an item characterized by two specific features (color and shape) within a group of items, each displaying only one of the two features. The items were presented for 600 ms. Performance was measured based on the number of correct trials and reaction time. The task included 20 trials. Brain regions involved in visual search include the primary visual cortex, the right posterior parietal cortex, the right dorsolateral prefrontal cortex, and the dorsal anterior cingulate cortex[4].

For each task, trial answers were partly designed by the platform itself and partly by the research team. Specifically, in the 2D mental rotation task, three trials were designed by www.gorilla.sc, while 13 were designed by the research team. For the visual search task, 12 trial stimuli were provided by www.gorilla.sc, while the researchers designed eight. In the dot-probe task, gorilla.sc provided stimuli for eight trials, while the researchers designed the eight remaining trials. Finally, in the 2-back task and the digit span text entry task the research team designed all the letter sequences as well as the digit sequences.

Participant data were excluded for each trial when reaction times were lower than 200ms or higher than 10,000ms. In such cases, only data from the relevant trial were excluded, not all the participant’s data. In addition, the dot-probe and 2-back tasks had implemented maximum time limits, leading to exclusion only because of too quick reaction times. For the dot-probe task, 2449 (20.05%) of the trials were excluded; for the 2-back task, 6119 (18.3%) of the trials were excluded; for the 2D mental rotation task, 1709 (10.2%) of the trials were excluded (458 (2.7%) for responding too quickly and 1252 (7.5%) for responding too slowly); for the visual search task, 4578 (12.8%) of the trials were excluded (3126 (8.7%) for reacting too quickly and 1452 (4.1%) for reacting too slowly); finally, for the digit span text entry task, 18,784 (17.7%) of the participant trials were excluded (16,598 (15.6%) for responding too quickly and 2186 (2.1%) for responding too slowly).

During the analysis of average drug dose and frequencies, participants who stated “0”, meaning that they had never taken that substance, for each respective substance, were excluded in order to get less skewed data.

**Supplementary Results**

In an exploratory analysis including the additional covariate “currently-using”, partial correlation testing revealed significant associations between recent psychedelic drug use and task accuracy across cognitive measures (dot-probe task: r = -0.232, p < 0.001; 2D mental rotation task: r = -0.267, p < 0.001; visual search task: r = -0.129, p < 0.006; 2-back task: r = -0.232, p < 0.001) The digit span text entry task revealed no significant association (r = -0.076, p = 0.112). Reaction time outcomes were also significantly associated with recent psychedelic use, showing a positive correlation for the dot-probe task (r = 0.214, p < 0.001) and a negative correlation for the 2D mental rotation task (r = -0.188, p < 0.001).

With respect to lifetime psychedelic use, significant negative correlations with task accuracy were observed for the dot-probe task (r = −0.116, p = 0.0145), the 2D mental rotation task (r = −0.125, p = 0.00874), and the 2-back task (r = −0.189, p < 0.001). The visual search task showed no significant correlation (r = 0.079, p = 0.099). In addition, lifetime use was negatively correlated with mean reaction time on the 2D mental rotation task (r = −0.154, p < 0.001). With regard to drug dose, in the dot-probe task reaction time outcomes were negatively correlated (r = -0.102, p = 0.033) as well as accuracy (r = -0.100, p = 0.035) in the 2-back task. Eight participants (1.05%) stated that they had never used a psychedelic substance across their lifetimes but subsequently selected at least one psychedelic substance from the list of possibly used psychoactive substances in the questionnaire. In the original analysis, we decided to treat those participants as non-users. However, to address this possibly inaccurate answer, we decided to exclude these eight participants in an additional sub-analysis. Because excluding the eight participants did not affect any statistical outcome, this analysis is not included here. All analyses presented in the manuscript and supplement thus reclassified the participants with inconsistent answers as described above.

Furthermore, it should be noted that the reported frequency of psychedelic substance use was often substantially higher than can be reasonably assumed, with some participants reporting use of more than 10 times per day during the past week or several hundred times across their lifetimes. Given that these assessments were based on self-report, such extreme values might reflect inaccurate or unreliable answers given by some participants.

**Supplementary References**

1. Koster EHW, Crombez G, Verschuere B, and De Houwer J. Selective attention to threat in the dot probe paradigm: differentiating vigilance and difficulty to disengage. *Behav Res Ther* (2004) 42: 1183-1192.

2. Vandenberg SG, and Kuse AR. Mental rotations, a group test of three-dimensional spatial visualization. *Percept Mot Skills* (1978) 47: 599-604.

3. Gogos A, Gavrilescu M, Davison S, Searle K, Adams J, Rossell SL, et al. Greater superior than inferior parietal lobule activation with increasing rotation angle during mental rotation: An fMRI study. *Neuropsychologia* (2010) 48: 529-535.

4. Bueichekú E, Miró‐Padilla A, and Ávila C. Resting‐state fMRI detects the effects of learning in short term: A visual search training study. *Hum Brain Mapp* (2019) 40: 2787-2799.

**Supplementary Table S1. Demographic and clinical characteristics**

| **Demographics** | **Total Participants N=759** |  |
| --- | --- | --- |
| **Age** |  |  |
| Median | 32 |  |
| Mean | 32.62 |  |
| Min-Max | 18-67 |  |
| Missing Age (NA) | 2 |  |
| **Sex** |  |  |
| Female – no. (%) | 205 (27.01) |  |
| Male – no.(%) | 554 (72.99) |  |
| **Nationality** |  |  |
| US American – no. (%) | 543 (71.5) |  |
| **Education** |  |  |
| Tertiary Education Degree – no. (%) | 608 (80.1) |  |
| **Psychiatric Condition** |  |  |
| Depression – no. (%) | 239 (31.49) |  |
| Self-reported addiction – no. (%) | 230 (30.30) |  |
| None – no. (%) | 199 (26.22) |  |
| ADHD – no. (%) | 110 (14.49) |  |
| Generalized anxiety disorder – no. (%) | 102 (13.44) |  |
| Any psychosis history – no. (%) | 94 (12.38) |  |
| Bipolar disorder – no. (%) | 86 (11.33) |  |
| Autism – no. (%) | 81 (10.67) |  |
| Social phobia – no. (%) | 61 (8.04) |  |
| PTSD – no. (%) | 60 (7.91) |  |
| Panic disorder – no. (%) | 57 (7.51) |  |
| Personality disorder – no. (%) | 51 (6.72) |  |
| HPPD – no. (%) | 45 (5.93) |  |
| Schizoaffective disorder – no. (%) | 33 (4.35) |  |
| Schizophrenia – no. (%) | 22 (2.90) |  |
| Other – no. (%) | 12 (1.58) |  |

Age in years; No.: Number;

| **Drug** | **Median Dose** | **Mean Dose** | **SD Dose** | **IQR Dose** | **Median Lifetime Use** | **Mean Lifetime Use** | **SD Lifetime Use** | **IQR Lifetime Use** | **Median Recent Use** | | **Mean Recent Use** | | **SD Recent Use** | | **IQR Recent Use** |  |
| --- | --- | --- | --- | --- | --- | --- | --- | --- | --- | --- | --- | --- | --- | --- | --- | --- |
| LSD | 125 | 144.64 | 100.34 | 166.75 | 133 | 265.33 | 276.26 | 489.5 | 23.0 | 30.94 | | 27.41 | | 49.00 | | |
| Psilocybin | 18 | 28.45 | 26.75 | 48 | 126.5 | 242.60 | 260.91 | 434.75 | 26.5 | 33.59 | | 28.54 | | 51.25 | | |
| Mescaline | 382 | 385.89 | 236.08 | 371 | 278.5 | 320.86 | 254.61 | 438.75 | 34.5 | 37.82 | | 26.88 | | 44.50 | | |
| DMT | 33.5 | 37.57 | 22.93 | 38 | 260.0 | 304.72 | 258.02 | 448.00 | 36.5 | 35.92 | | 26.13 | | 48.00 | | |
| 5-MeO-DMT | 39.5 | 40.07 | 25.29 | 47 | 330.0 | 361.29 | 255.40 | 424.50 | 37.0 | 39.27 | | 25.64 | | 36.50 | | |
| Ibogaine | 37 | 39.22 | 24.3 | 41 | 345.0 | 361.32 | 245.20 | 406.50 | 29.0 | 36.61 | | 23.70 | | 37.00 | | |
| DOI | 4 | 4.45 | 2.43 | 4 | 372.0 | 390.11 | 259.80 | 447.75 | 37.5 | 42.21 | | 27.67 | | 51.75 | | |
| MDMA | 100 | 94.56 | 51.52 | 80 | 177.0 | 276.39 | 262.45 | 446.50 | 28.0 | 38.36 | | 30.85 | | 53.00 | | |
| Scopolamine | 43.5 | 42.81 | 25.37 | 42 | 396.0 | 398.50 | 259.61 | 449.00 | 35.5 | 42.61 | | 29.31 | | 50.00 | | |
| Salvia | 441.5 | 418.63 | 241.33 | 406.25 | 268.5 | 318.39 | 261.65 | 424.25 | 32.0 | 35.77 | | 25.21 | | 38.00 | | |
| Ketamine | 66 | 75.18 | 48.78 | 80 | 240.0 | 301.56 | 263.40 | 454.50 | 27.0 | 31.39 | | 24.51 | | 30.00 | | |
| PCP | 22 | 21.06 | 12.57 | 23 | 381.0 | 392.35 | 257.46 | 446.25 | 40.5 | 39.63 | | 26.03 | | 45.00 | | |

**Supplementary Table S2: Assessment of classic psychedelic drug use and use of substances with hallucinogenic properties**

Drug assessment of classic psychedelics and substances with hallucinogenic properties including median dose (participants had to state the median dose out of all doses ever taken) and mean dose (mean dose out of all doses ever ingested). SD: Standard deviation; IQR: Interquartile range; LSD: lysergic acid diethylamide; DMT: N,N-dimethyltryptamine (The term “DMT” refers to ayahuasca and is equivalent to “DMT+Harmine (see Figure 2B in the manuscript)); 5-MeO-DMT: tryptamine; DOI: 2,5-Dimethoxy-4-iodoamphetamine; MDMA: methylenedioxymethamphetamine; PCP: phencyclidine; Lifetime use: frequency of drug use accumulated over lifetime; Recent use: frequency of use in the week prior to study participation. The dose of LSD was given in mcg; the dose of psilocybin were given in g (participants had to state weight of dried mushrooms); doses of mescaline, DMT, 5-Me0-DMT, ibogaine, DOI, MDMA, scopolamine, salvia, ketamine, and PCP were given in mg.

**Supplementary Table S3. Descriptive results of 10 main outcome parameters (accuracy and mean reaction time) for the five cognitive tasks completed by non-users, lifetime users, and recent users.**

| **Variable** | **Non-user** | **Lifetime user** | **Recent user** | **Median Non-user** | **Median Lifetime user** | **Median Recent user** | **Min Non-user** | **Min Lifetime-user** | **Min Recent-user** | | **Max Non-user** | **Max Lifetime-user** | **Max Recent user** |
| --- | --- | --- | --- | --- | --- | --- | --- | --- | --- | --- | --- | --- | --- |
| Accuracy dot-probe | 144 | 181 | 434 | 13.00 (7.00 - 16.00) | 16.00 (15.00 - 16.00) | 9.00 (4.00 - 15.00) | 1 | 1 | | 1 | 16 | 16 | 16 |
| Accuracy Digit span | 144 | 181 | 434 | 4.5 (3.00 - 7.00) | 6.00 (4.00 - 7.00) | 4.00 (3.00 - 7.00) | 1 | 1 | | 1 | 9 | 10 | 10 |
| Accuracy 2-Back | 144 | 181 | 434 | 15.00 (8.75 - 23.00) | 25.00 (18.00 - 30.00) | 9.00 (3.00 - 17.00) | 1 | 1 | | 1 | 34 | 35 | 34 |
| Accuracy 2D-Rotation | 144 | 181 | 434 | 8.00 (5.750 - 10.00) | 11.00 (9.00 - 12.00) | 6.00 (4.00 - 9.00) | 1 | 3 | | 1 | 16 | 15 | 16 |
| Accuracy Visual Search | 144 | 181 | 434 | 10.00 (7.00 - 15.00) | 17.00 (10.00 - 19.00) | 9.00 (5.00 - 11.00) | 1 | 1 | | 1 | 20 | 20 | 20 |
| Reaction Time Digit span | 144 | 181 | 434 | 4252.210 (3574.12 - 5038.1) | 3987.12 (3266.2 - 4749.75) | 4076.65 (3222.18 - 5499.64) | 1562.8 | 2206.7 | | 1690 | 9869.4 | 8307.4 | 9820.7 |
| Reaction Time Dot-probe | 144 | 181 | 434 | 721 (448.16 - 998.56) | 411.07 (366.33 - 481.46) | 690.24 (448.67 - 951.22) | 202.8 | 242.7 | | 200.1 | 2795.5 | 1786.8 | 3375.5 |
| Reaction Time 2-Back | 144 | 181 | 434 | 830.94 (689.87 - 1048.36) | 793.74 (702.21 - 939.93) | 803.22 (564.94 - 991.69) | 203.3 | 416.45 | | 203.9 | 1473.9 | 1706.7 | 1873.4 |
| Reaction Time 2D-Rotation | 144 | 181 | 434 | 2636.41 (1668.33 - 3342.69) | 2971.64 (2411.75 - 3685.21) | 1982.9 (957.9 - 2891.21) | 215.55 | 655.775 | | 213.65 | 5735.182 | 6119.817 | 8406 |
| Reaction Time Visual Search | 144 | 181 | 434 | 919.06 (620.5 - 1379.56) | 918.92 (746.47 - 1156.1) | 947.57 (630.84 - 1441.27) | 206.5667 | 323.5 | | 203.5 | 3592.389 | 4490.867 | 4104.833 |

| **Supplementary Table S4. Recent drug use x cognitive tasks** | | | | | | |  |
| --- | --- | --- | --- | --- | --- | --- | --- |
| **Task** | **Variable** | **Estimate** | ***P* Value** | | **Test Statistic** | |  |
| Dot-probe | Reaction Time | 0.258 | < 0.001 | | 5.594 | |  |
| Dot-probe | Accuracy | -0.304 | < 0.001 | | -6.697 | |  |
| 2D-Rotation | Reaction Time | -0.232 | < 0.001 | | -4.998 | |  |
| 2D-Rotation | Accuracy | -0.321 | < 0.001 | | -7.394 | |  |
| Visual Search | Reaction Time | -0.009 | 0.851 | | -0.188 | |  |
| Visual Search | Accuracy | -0.161 | 0.000659 | | -3.431 | |  |
| 2-Back | Reaction Time | 0.01 | 0.839 | | 0.203 | |  |
| 2-Back | Accuracy | -0.305 | < 0.001 | | -6.726 | |  |
| Digit Span | Reaction Time | 0.1 | 0.840 | | 0.202 | |  |
| Digit Span | Accuracy | -0.097 | 0.0424 | | -2.035 | |  |
| **Lifetime Drug Use x Cognitive Tasks** | | | |  | |  | |
| **Task** | **Variable** | **Estimate** | ***P* Value** | | **Test Statistic** | |  |
| Dot-probe | Reaction Time | 0.067 | 0.159 | | 1.411 | |  |
| Dot-probe | Accuracy | -0.154 | 0.00119 | | -3.262 | |  |
| Rotation | Reaction Time | -0.176 | 0.000194 | | -3.758 | |  |
| Rotation | Accuracy | -0.160 | 0.000739 | | -3.399 | |  |
| Visual Search | Reaction Time | -0.001 | 0.979 | | -0.027 | |  |
| Visual Search | Accuracy | -0.096 | 0.0436 | | -2.023 | |  |
| 2-Back | Reaction Time | -0.034 | 0.482 | | -0.704 | |  |
| 2-Back | Accuracy | -0.223 | < 0.0001 | | -4.801 | |  |
| Digit Span | Reaction Time | 0.012 | 0.809 | | 0.241 | |  |
| Digit Span | Accuracy | -0.002 | 0.962 | | -0.048 | |  |

Above: Partial Spearman Correlations of consumed psychedelics and substances with hallucinogenic properties in the week prior to task performance (recent use) and outcomes (accuracy and mean reaction time) of five cognitive tasks (dot-probe task, 2D mental rotation task, visual search task, 2-back task, digit span text entry task). Coefficients between recent psychedelic use and cognitive performance, controlling for age at first use, sex, education level, age, use of non psychedelic drugs, and psychiatric diagnosis. Positive estimates indicate associations with slower reaction times and higher accuracy, while negative estimates indicate lower accuracy or faster responses. Significant correlations (p < 0.05) suggest potential negative cognitive effects for accuracy across all five tasks. Below: Same as above but for participants who stated taking any psychedelic drug or substance with hallucinogenic properties in their lifetime (lifetime users). Significant correlations were found for negative effects on accuracy for the dot-probe, 2D mental rotation, visual search, and 2-back tasks. Correlations for mean reaction time were mixed.

| **LSD** |  |  |  | **Psilocybin** |  |  |  |
| --- | --- | --- | --- | --- | --- | --- | --- |
| **Task** | **Variable** | **Spearman** | ***P* value** | **Task** | **Variable** | **Spearman** | ***P* value** |
| Dot-probe | Accuracy | -0.176 | 0.0000332 | Dot-probe | Accuracy | -0.519 | < 0.001 |
| Dot-probe | Reaction Time | 0.0870 | 0.0408 | Dot-probe | Reaction Time | 0.324 | < 0.001 |
| 2D-Rotation | Accuracy | -0.183 | 0.00000520 | 2D-Rotation | Accuracy | -0.502 | < 0.001 |
| 2D-Rotation | Reaction Time | -0.155 | 0.000110 | 2D-Rotation | Reaction Time | -0.399 | < 0.001 |
| Visual Search | Accuracy | -0.144 | 0.000532 | Visual Search | Accuracy | -0.433 | < 0.001 |
| Visual Search | Reaction Time | -0.00537 | 0.898 | Visual Search | Reaction Time | -0.0158 | 0.705 |
| 2-Back | Accuracy | -0.241 | 0.00000000731 | 2-Back | Accuracy | -0.551 | < 0.001 |
| 2-Back | Reaction Time | -0.0180 | 0.671 | 2-Back | Reaction Time | -0.0317 | 0.454 |
| Digit Span | Accuracy | -0.106 | 0.0137 | Digit Span | Accuracy | -0.256 | < 0.001 |
| Digit Span | Reaction Time | 0.0258 | 0.548 | Digit Span | Reaction Time | 0.0822 | 0.0554 |
|  |  |  |  |  |  |  |  |
| **Alcohol** |  |  |  | **THC** |  |  |  |
| **Task** | **Variable** | **Spearman** | ***P* value** | **Task** | **Variable** | **Spearman** | ***P* value** |
| Dot-probe | Accuracy | -0.497 | < 0.001 | Dot-probe | Accuracy | -0.420 | < 0.001 |
| Dot-probe | Reaction Time | 0.348 | < 0.001 | Dot-probe | Reaction Time | 0.212 | < 0.001 |
| 2D-Rotation | Accuracy | -0.470 | < 0.001 | 2D-Rotation | Accuracy | -0.376 | < 0.001 |
| 2D-Rotation | Reaction Time | -0.321 | < 0.001 | 2D-Rotation | Reaction Time | -0.306 | < 0.001 |
| Visual Search | Accuracy | -0.384 | < 0.001 | Visual Search | Accuracy | -0.337 | < 0.001 |
| Visual Search | Reaction Time | -0.0103 | 0.783 | Visual Search | Reaction Time | -0.0277 | 0.459 |
| 2-Back | Accuracy | -0.467 | < 0.001 | 2-Back | Accuracy | -0.413 | < 0.001 |
| 2-Back | Reaction Time | -0.00774 | 0.839 | 2-Back | Reaction Time | -0.0827 | 0.03 |
| Digit Span | Accuracy | -0.272 | < 0.001 | Digit Span | Accuracy | -0.199 | < 0.001 |
| Digit Span | Reaction Time | 0.0711 | 0.067 | Digit Span | Reaction Time | 0.0398 | 0.306 |

**Supplementary Table S5: Corrected Spearman correlations for dose-dependent accuracy and reaction time outcomes for LSD, psilocybin, alcohol and THC**

Correlations were calculated separately for LSD, psilocybin, alcohol, and THC, and for each of five cognitive tasks (dot-probe, 2D mental rotation, visual search, 2-back, and digit span text entry), assessing accuracy and reaction time. Spearman’s rho values and corresponding Bonferroni-corrected *p*-values are reported. Dose-dependent effects were observed for LSD, psilocybin, alcohol, and THC for accuracy across all five cognitive tasks, with higher median doses correlated with lower accuracy; outcomes for reaction time varied.

**Supplementary Table S6. Descriptive results for four mental health questionnaires grouped by non-user, lifetime user, and recent user**

| **Questionnaire** | **Non-user** | **Lifetime user** | | **Recent**  **user** | **Median Non-user** | | **Median Lifetime user** | | **Median recent user** | | **Min Non-user** | | **Min**  **Lifetime user** | | **Min Recent user** | | **Max Non-user** | | **Max Lifetime user** | | **Max Recent user** | |
| --- | --- | --- | --- | --- | --- | --- | --- | --- | --- | --- | --- | --- | --- | --- | --- | --- | --- | --- | --- | --- | --- | --- |
| DES-B | 144 | 181 | 434 | | 12.00 (5.00 - 20.25) | 5.00 (4.00 - 10.00) | | 21.00 (17.00 - 25.00) | | 0 | | 0 | | 0 | | 28 | | 26 | | 32 | |  |
| HPPD | 144 | 181 | 434 | | 10.00 (3.00 - 20.00) | 3.00 (1.00 - 6.00) | | 24.00 (18.00 - 28.00) | | 0 | | 0 | | 0 | | 32 | | 26 | | 36 | |  |
| PHQ-9 | 144 | 181 | 434 | | 11.00 (4.00 - 15.25) | 5.00 (2.00 - 10.00) | | 16.00 (13.00 - 20.00) | | 0 | | 0 | | 0 | | 25 | | 23 | | 26 | |  |
| WHOQOL-BREF-Overall | 144 | 181 | 434 | | 16.00 (14.00 - 18.00) | 16.00 (12.00 - 18.00) | | 16.00 (14.00 - 18.00) | | 6 | | 6 | | 4 | | 20 | | 20 | | 20 | |  |
| Environmental Domain | 144 | 181 | 434 | | 15.75 (14.00 - 17.00) | 15.50 (14.00 - 17.00) | | 15.50 (14.00 - 17.00) | | 10 | | 7.5 | | 7 | | 20 | | 20 | | 20 | |  |
| Physical Domain | 144 | 181 | 434 | | 14.29 (13.14 - 15.43) | 14.86 (13.14 - 17.14) | | 13.71 (13.14 - 14.86) | | 6.86 | | 8.57 | | 8.57 | | 20 | | 20 | | 20 | |  |
| Psychological Domain | 144 | 181 | 434 | | 14.33 (13.17 - 16.17) | 14.00 (12.00 - 15.33) | | 17.67 (13.33 - 15.33) | | 5.33 | | 5.33 | | 6.67 | | 20 | | 20 | | 19.33 | |  |
| Social Domain | 144 | 181 | 434 | | 16.00 (13.33 - 17.33) | 13.33 (10.67 - 16.00) | | 16.00 (13.33 - 17.33) | | 4 | | 4 | | 4 | | 20 | | 20 | | 20 | |  |

DES-B: Brief Dissociative Experiences Scale (highest possible score: 32; higher score indicates more intense/frequent dissociative symptoms; HPPD: Hallucinogen Persisting Perception Disorder (highest possible score: 36, higher score indicates more intense/frequent HPPD symptoms); PHQ-9: nine-item Patient Health Questionnaire (highest possible score: 27, higher score indicates more severe depressive symptoms); WHOQOL-BREF: World Health Organization - Quality of Life questionnaire. The WHOQOL-BREF questionnaire is divided into five parts: overall quality of life and physical, psychological, social, and environmental domains (highest score for each domain: 20, higher scores indicate higher quality of life in each domain).

**Supplementary Table S7: Explorative analysis: Kruskal-Wallis test for five cognitive tasks and Mental Health Questionnaires excluding “currently-using” participants in the lifetime user group**

| **Dependent Variable** | **Test Statistic** | ***p*-Value** |
| --- | --- | --- |
| Reaction Time Dot-probe | 110.301 | < 0.001 |
| Accuracy Dot-probe | 152.555 | < 0.001 |
| Reaction Time 2D-Rotation | 109.788 | < 0.001 |
| Accuracy 2D-Rotation | 189.175 | < 0.001 |
| Reaction Time Visual Search | 0.576 | 0.7499 |
| Accuracy Visual Search | 84.847 | < 0.001 |
| Reaction Time 2-back | 4.285 | 0.1173 |
| Accuracy 2-back | 147.051 | < 0.001 |
| Reaction Time Digit span | 4.226 | 0.1209 |
| Accuracy Digit span | 37.570 | < 0.001 |
| PHQ-9 | 200.642 | < 0.001 |
| HPPD | 258.724 | < 0.001 |
| Physical Domain | 57.082 | < 0.001 |
| Psychological Domain | 12.116 | 0.0023 |
| Social Domain | 43.683 | < 0.001 |
| Environmental Domain | 0.248 | 0.8836 |
| WHOQOL-BREF-Overall | 11.53 | 0.0031 |
| DES-B | 240.302 | < 0.001 |

Exploratory analysis of Kruskal-Wallis test results comparing cognitive and mental health outcomes between three groups based on psychedelic use (use within last week, lifetime use, and never used). Participants stating lifetime use and that they were under the influence of a psychoactive drug during study participation, but did not state psychedelic drug use in the week prior to study participation (n=23) and were thus part of the lifetime users group were excluded from the analysis. The test statistics (χ²) and corresponding p-values are reported. Significant results (p < .05) indicate group differences for the respective outcome variables.

**Supplementary Table S8: Spearman Correlation of lifetime drug use (excluding “currently-using” users of the lifetime group) x cognitive tasks**

| **Task** | **Variable** | **Estimate** | ***P*-Value** | **Statistic** |
| --- | --- | --- | --- | --- |
| Dot-probe | Mean Reaction Time | 0.079 | 0.106 | 1.620 |
| Dot-probe | Accuracy | -0.245 | < 0.001 | -5.192 |
| Rotation | Mean Reaction Time | -0.228 | < 0.001 | -4.811 |
| Rotation | Accuracy | -0.240 | < 0.001 | -5.073 |
| Visual Search | Mean Reaction Time | -0.021 | 0.663 | -0.436 |
| Visual Search | Accuracy | -0.138 | < 0.001 | -2.856 |
| 2-back | Mean Reaction Time | -0.064 | 0.188 | -1.320 |
| 2-back | Accuracy | -0.277 | < 0.001 | -5.921 |
| Digit Span | Mean Reaction Time | 0.014 | 0.772 | 0.290 |
| Digit Span | Accuracy | -0.043 | 0.374 | -0.889 |

Exploratory analysis: Partial Spearman correlation analyses between lifetime use of hallucinogens and cognitive outcome measures, controlling for covariates (age at first use, sex, education level, current age, use of non-hallucinogens, and psychiatric diagnosis). Participants stating lifetime use and that they were under the influence of a psychoactive drug during study participation, but did not state psychedelic drug use in the week prior to study participation (n=23) and were thus part of the lifetime users group were excluded from the analysis. Correlation estimates, test statistics, and p-values are reported. Significant negative correlations indicate worse cognitive performance with increasing lifetime hallucinogen use.

**Supplementary Table S9: Exploratory analysis: Kruskal-Wallis test for five cognitive tasks and Mental Health Questionnaires excluding all “currently-using” participants**

| **Dependent Variable** | **Test Statistic** | ***p*-Value** |
| --- | --- | --- |
| Reaction Time Dot-probe | 77.472 | < 0.001 |
| Accuracy Dot-probe | 65.458 | < 0.001 |
| Reaction Time 2D-Rotation | 49.010 | < 0.001 |
| Accuracy 2D-Rotation | 99.214 | < 0.001 |
| Reaction Time Visual Search | 5.037 | 0.0806 |
| Accuracy Visual Search | 39.147 | < 0.001 |
| Reaction Time 2-back | 4.117 | 0.1276 |
| Accuracy 2-back | 56.276 | < 0.001 |
| Reaction Time Digit span | 4.581 | 0.1012 |
| Accuracy Digit span | 19.581 | < 0.001 |
| PHQ-9 | 57.884 | < 0.001 |
| HPPD | 86.341 | < 0.001 |
| Physical Domain | 22.630 | < 0.001 |
| Psychological Domain | 9.061 | 0.0108 |
| Social Domain | 27.881 | < 0.001 |
| Environmental Domain | 1.856 | 0.3953 |
| WHOQOL-BREF-Overall | 9.400 | 0.0091 |
| DES-B | 79.961 | < 0.001 |

Exploratory analysis of Kruskal-Wallis test results comparing cognitive and mental health outcomes between three groups based on psychedelic use (use within last week, lifetime use, and never used). All participants stating that they were under the influence of a psychoactive drug during assessment were excluded from analysis. The test statistics (χ²) and corresponding p-values are reported. Significant results (p < .05) indicate group differences for the respective outcome variables. Non-users: n = 144; Lifetime users n = 158; Recent users n = 156.

**Supplementary Table S10: Exploratory Analysis: Post-hoc Dunn’s test for five cognitive tasks and Mental Health Questionnaires excluding all “currently-using” participants**

| **Variable** | **Comparison** | **Z** | **P.adj** |
| --- | --- | --- | --- |
| Reaction Time Dot-probe | Non-user vs Lifetime user | 8.5572 | < 0.001 |
| Reaction Time Dot-probe | Non-user vs Recent user | 2.7766 | 0.0165 |
| Reaction Time Dot-probe | Lifetime user vs Recent user | -5.7550 | < 0.001 |
| Accuracy Dot-probe | Non-user vs Lifetime user | -7.2349 | < 0.001 |
| Accuracy Dot-probe | Non-user vs Recent user | -0.7528 | 1 |
| Accuracy Dot-probe | Lifetime user vs Recent user | 6.5356 | < 0.001 |
| Reaction Time 2D-Rotation | Non-user vs Lifetime user | -4.4406 | < 0.001 |
| Reaction Time 2D-Rotation | Non-user vs Recent user | 2.3077 | 0.0630 |
| Reaction Time 2D-Rotation | Lifetime user vs Recent user | 6.8880 | < 0.001 |
| Accuracy 2D-Rotation | Non-user vs Lifetime user | -8.6258 | < 0.001 |
| Accuracy 2D-Rotation | Non-user vs Recent user | -0.2400 | 1 |
| Accuracy 2D-Rotation | Lifetime user vs Recent user | 8.5448 | < 0.001 |
|  |  |  |  |
|  |  |  |  |
|  |  |  |  |
| Accuracy Visual Search | Non-user vs Lifetime user | -5.3831 | < 0.001 |
| Accuracy Visual Search | Non-user vs Recent user | -0.0290 | 1 |
| Accuracy Visual Search | Lifetime user vs Recent user | 5.3636 | < 0.001 |
| Accuracy 2-back | Non-user vs Lifetime user | -6.8286 | < 0.001 |
| Accuracy 2-back | Non-user vs Recent user | -0.9737 | 0.9906 |
| Accuracy 2-back | Lifetime user vs Recent user | 5.9073 | < 0.001 |
| Accuracy Digit Span | Non-user vs Lifetime user | -4.3326 | < 0.001 |
| Accuracy Digit Span | Non-user vs Recent user | -1.6280 | 0.3106 |
| Accuracy Digit Span | Lifetime user vs Recent user | 2.7754 | 0.0036 |
| PHQ-9 | Non-user vs Lifetime user | 5.3055 | < 0.001 |
| PHQ-9 | Non-user vs Recent user | -1.8853 | 0.1782 |
| PHQ-9 | Lifetime user vs Recent user | -7.3459 | < 0.001 |
| HPPD | Non-user vs Lifetime user | 7.0489 | < 0.001 |
| HPPD | Non-user vs Recent user | -1.4889 | 0.4096 |
| HPPD | Lifetime user vs Recent user | -8.7196 | < 0.001 |
| Physical Domain | Non-user vs Lifetime user | -3.812 | 0.0004 |
| Physical Domain | Non-user vs Recent user | 0.4396 | 1 |
| Physical Domain | Lifetime user vs Recent user | 4.3413 | < 0.001 |
| Psychological Domain | Non-user vs Lifetime user | 2.9457 | 0.0097 |
| Psychological Domain | Non-user vs Recent user | 1.0074 | 0.9413 |
| Psychological Domain | Lifetime user vs Recent user | -1.9754 | 0.1447 |
| Social Domain | Non-user vs Lifetime user | 5.2406 | < 0.001 |
| Social Domain | Non-user vs Recent user | 2.1824 | 0.0872 |
| Social Domain | Lifetime user vs Recent user | -3.1148 | 0.0055 |
| WHOQOL-BREF-Overall | Non-user vs Lifetime user | 2.9866 | 0.0085 |
| WHOQOL-BREF-Overall | Non-user vs Recent user | 0.9666 | 1 |
| WHOQOL-BREF-Overall | Lifetime user vs Recent user | -2.0589 | 0.1185 |
| DES-B | Non-user vs Lifetime user | 6.4925 | < 0.001 |
| DES-B | Non-user vs Recent user | -1.8597 | 0.1888 |
| DES-B | Lifetime user vs Recent user | -8.5314 | < 0.001 |

Post-hoc Dunn’s test following a Kruskal–Wallis test. Comparisons are presented between non-users (n = 144), lifetime users (n = 158), and recent users (n = 156). Z-values and adjusted p-values (Bonferroni correction) are reported for each variable.

**Supplementary Figure S1. Additional Variables of Psychedelic Use.**

**A**: The frequency of different settings in which participants reported psychedelic use. **B**: Age at first psychedelic drug use. **C**: Number of psychiatric inpatient care instances. Most participants had never been hospitalized. **D**: Proportion of personality disorder diagnoses among participants.

**Supplementary Figure S2. Partial Spearman Correlation between drug use and cognitive performance**

**A:** Correlation between total lifetime drug use and cognitive task outcomes (task accuracy and reaction time) across five cognitive tasks: digit span text entry, dot-probe, 2-back, 2D-rotation, and visual search. **B:** Correlation between recent (within the week prior to study participation) total drug use and cognitive task outcomes (task accuracy and reaction time). All plots depict partial Spearman correlation coefficients controlling for age, sex, education, use of non-hallucinogens, psychiatric diagnosis, and age at first use. Blue regression lines represent the direction of the correlation trend, with shaded areas indicating 95% confidence interval.
